# A nuclear localization for Avr2 from *Fusarium oxysporum* is required to activate the tomato resistance protein I-2

**DOI:** 10.3389/fpls.2013.00094

**Published:** 2013-04-11

**Authors:** Lisong Ma, Ben J. C. Cornelissen, Frank L. W. Takken

**Affiliations:** Molecular Plant Pathology, Swammerdam Institute for Life Sciences, University of AmsterdamAmsterdam, Netherlands

**Keywords:** disease resistance, effector, Avr2, *Fusarium oxysporum*, tomato, I-2

## Abstract

Plant pathogens secrete effector proteins to promote host colonization. During infection of tomato xylem vessels, *Fusarium oxysporum* f. sp. *lycopersici* (*Fol*) secretes the Avr2 effector protein. Besides being a virulence factor, Avr2 is recognized intracellularly by the tomato I-2 resistance protein, resulting in the induction of host defenses. Here, we show that *AVR2* is highly expressed in root- and xylem-colonizing hyphae three days post inoculation of roots. Co-expression of *I-2* with *AVR2* deletion constructs using agroinfiltration in *Nicotiana benthamiana* leaves revealed that, except for the N-terminal 17 amino acids, the entire *AVR2* protein is required to trigger *I-2*-mediated cell death. The truncated Avr2 variants are still able to form homo-dimers, showing that the central region of Avr2 is required for dimerization. Simultaneous production of I-2 and Avr2 chimeras carrying various subcellular localization signals in *N. benthamiana* leaves revealed that a nuclear localization of Avr2 is required to trigger I-2-dependent cell death. Nuclear exclusion of Avr2 prevented its activation of I-2, suggesting that Avr2 is recognized by I-2 in the nucleus.

## Introduction

Many plant pathogens employ small, secreted proteins called effectors, to facilitate infection and to establish disease (Ellis et al., 2009; Tyler, 2009). Effectors interfere with biological processes of the host to the benefit of the pathogen (Kamoun, 2006; Alfano, 2009). To counteract pathogens, plants evolved resistance (R) proteins to perceive the presence or actions of these effectors (Chisholm et al., 2006; Maekawa et al., 2011). Although some R proteins are cell-surface receptors, most of them are cytosolic proteins of the nucleotide-binding leucine-rich repeat (NLR) type. Effector perception leads to activation of “effector-triggered immunity” (ETI), a response that is typically associated with programmed cell death of the infected cells. The induced resistance responses restrict outgrowth of the pathogen from the infection site (Spoel and Dong, 2012).

Many bacterial pathogens, such as *Pseudomonas syringae*, employ a type III secretion system to directly deliver effectors into the cytosol of plant cells (Shames and Finlay, 2012). Concomitantly, most *R* genes controlling bacterial pathogens encode NLR immune receptors with a predicted cytosolic location. Plant pathogenic fungi and oomycetes lack a type III secretion system and they secrete their effectors directly into intercellular spaces such as the apoplast or the xylem sap. Although some resistance genes controlling pathogenic fungi encode extracellular immune receptors, such as the Cf and Ve proteins controlling, respectively, *Cladosporium fulvum* and *Verticillium dahliae* (Thomma et al., 2011), most encode intracellular receptors (Dodds and Rathjen, 2010). Resistance to haustorium-forming pathogens is typically conferred by cytosolic receptors (Maekawa et al., 2011). The effectors are secreted into the periplasmic space surrounding the haustorium, from which a subset is taken up by the host cell, allowing intracellular perception (Whisson et al., 2007; Dou et al., 2008; Rafiqi et al., 2010; Schornack et al., 2010; De Jonge et al., 2011). The conserved RxLR motif found in many oomycete effectors is likely involved in the uptake process, as mutations in this motif abolish uptake (Grouffaud et al., 2008). Resistance to xylem-colonizing fungal pathogens that do not form haustoria can also be mediated by cytosolic NLR resistance genes, suggesting the uptake of the corresponding effector from the xylem sap by the host cells. The mechanism by which these fungal effectors enter the host cell, and the subcellular localization where they are perceived by the host immune receptor, are as yet unknown.

The interaction between tomato and the xylem-colonizing fungus *Fusarium oxysporum* f. sp. *lycopersici* (*Fol*) has emerged as a model system to study NLR-mediated recognition of xylem secreted effectors (Takken and Rep, 2010). *Fol* is a soil born pathogen that causes vascular wilt disease by colonizing the xylem vessels of roots and stems (Michielse and Rep, 2009). Resistance to *Fol* in tomato is conferred by so-called “immunity” or “*I*” genes, and three of these genes have been introgressed from wild *Solanum* relatives into commercial varieties: *I* (or *I-1*), *I-2*, and *I-3*. *I-2* has been cloned and encodes a classical NLR protein that mediates resistance upon recognition of the Avr2 effector protein from *Fol* (Simons et al., 1998; Houterman et al., 2009). *I-2* promoter-reporter studies revealed that the gene is specifically expressed in the parenchyma cells adjacent to the xylem vessels, but the subcellular localization of I-2 is unknown (Mes et al., 2000). Typically, ETI induces a programmed cell death response. However, *I-2*-mediated resistance seems to be distinct, as *Fol* recognition triggers specific responses in the parenchymal cells, which include accumulation of phenolics, callose deposition, and formation tyloses (outgrowth of xylem contact cells) and gels in the infected vessels, but not cell death (Beckman, 2000; Takken and Rep, 2010).

Xylem sap proteomics of *Fol* infected tomato resulted in identification of the *Fol* Avr2 protein. The *AVR2* gene encodes a 15.7 kDa mature protein (after cleavage of the N-terminal signal peptide), without discernable sequence similarity to other proteins (Houterman et al., 2009). Avr2 is not only an avirulence determinant of *I-2*, it is also a virulence factor required for full virulence of the fungus on susceptible plants. Race 3 *Fol* strains that can overcome *I-2* carry amino acid substitutions in Avr2 that prevent its recognition by I-2 while retaining its virulence function (Houterman et al., 2009). Whereas I-2-mediated resistance typically does not involve a cell death response, such as response is induced upon co-expression of *AVR2* and *I-2* in *Nicotiana benthamiana* using agroinfiltration or upon Potato Virus X-mediated expression of *AVR2* in *I-2* tomato. The strongest cell death response was found upon expression of a truncated *AVR2* variant that is not secreted by the transformed host cells (Houterman et al., 2009). This potentiated response implies intracellular recognition of Avr2 by I-2 and suggests that during natural infection the effector is taken up from the xylem sap by the adjacent plant cells (Houterman et al., 2009).

To determine where in the plant Avr2 is being produced by the fungus, allowing its perception by I-2, we studied the *in planta* expression of *AVR2* during infection. Since Avr2 is perceived intracellularly by I-2, we also examined its subcellular localization and determined the subcellular localization where Avr2 activates I-2. Finally, Avr2 deletion studies were performed to identify the minimal region that is required for dimerization and I-2 activation.

## Materials and methods

### Generation of transgenic *Fol* strains

Homologous recombination was used to replace the *AVR2* gene with a cassette containing the gene of interest and a hygromycin resistance gene. To generate the *AVR2*-promoter-*RFP* construct, the terminator of *AVR2* gene was PCR amplified with primer combination FP2708/FP2663 listed in Table A1 using *Fol*007 genomic DNA as template. The resulting amplicon was cloned into the *Kpn*I site of pRW2h:Δ*AVR2*. In this vector the hygromycin resistance gene cassette is flanked by 1266 bp and 717 bp of sequences upstream and downstream of the *AVR2* ORF (Houterman et al., 2009). The correct orientation of the *AVR2* terminator was confirmed by PCR using primer set FP1074/FP2663. The pRW2h: Δ*AVR2*-T vector was generated. RFP was amplified from the pGWB454 plasmid DNA using primer set FP2706/FP2707 listed in Table A1 (Nakagawa et al., 2007). The obtained fragments were digested with *Spe*I, gel purified, and ligated into a *Spe*I digested pRW2h: Δ*AVR2*-T vector containing the *AVR2* terminator. The orientation of *RFP* constructs was confirmed by PCR with primer set FP1074/FP2707. The obtained plasmid pRW2h:*pAVR2:RFP* was transformed into *Agrobacterium tumefaciens* EHA105 and used for subsequent *A. tumefaciens*-mediated *Fol* transformation according to Rep et al. (2004). *Fol* transformants capable of growing on 100 μg mL^−1^ hygromycin (Duchefa) were checked by PCR for the absence of the *AVR2* gene using primer pair FP1074/FP965. Presence of the right and left borders of these constructs was confirmed with primers annealing just outside the flanking sequences, these were FP745/FP1075 (right border) and FP659/FP1166 (left border), respectively. Out of the 150 hygromycin resistant transformants one genuine *AVR2* replacement mutant was identified based on the absence of *AVR2* and the presence of Monomeric red fluorescent protein (mRFP) and the hygromycin cassette in the *AVR2* locus (data not shown).

### Vector construction

For localization studies, the pENTR207:Δ*spAVR2* or pENTR207:*AVR2* plasmid, described previously (Houterman et al., 2009), was used to recombine *AVR2* or Δ*spAVR2* into binary vector pGWB454 and pGWB451 (Nakagawa et al., 2007) according to the Gateway protocol for LR recombination reaction (Invitrogen). In the pGWB454 constructs Avr2 is fused to an RFP tag present in the vector. In the construct pGWB451:Δ*AVR2* Avr2 is fused to a GFP tag present in the vector. To construct *NLS*-Δ*spAVR2*:*GFP*, a nuclear localization signal (NLS) was introduced into forward primer FP2959 and the fragment was amplified together with reverse primer FP2222 from the pGWB451:Δ*AVR2* plasmid. To construct Δ*spAVR2-NES: GFP*, part of the nuclear export signal (NES) was introduced into the reverse primer FP3483 and the fragment was amplified together with forward primer FP2525. The fragment obtained with this primer set was used as template for a second round of PCR using primer set FP2525/FP3482. To create *CBL-ΔspAVR2-NES:GFP*, first part of the myristoylation signal (CBL) (Batistic et al., 2008) was introduced into forward primer FP3479 and part of the NES was introduced into the reverse primer FP3483. The fragment obtained with this primer set was used as template for a second round of PCR using primer set FP3478/FP3482. The resulted fragment contained the complete CBL coding sequences in the N-terminus and a NES coding sequence in the C-terminus of *AVR2*. The fragment harboring the mutated CBL and NES coding sequences was generated using the same strategy but by using primer sets FP3481/FP3485 and FP3480/FP3484, respectively. Finally the four amplicons were digested with *Xba*I and *Sac*I, and ligated into pGWB451 digested with the same enzymes.

Three primer combinations: FP2684/FP1751, FP2699/FP1751, and FP1749/FP2685 were used to amplify truncated *AVR2* fragments from CTAPi:Δ*spAVR2*. Subsequently, gateway *attB* linkers were added via PCR using primers FP872 and FP873. The obtained PCR products were introduced into entry clone pDONR207 (Invitrogen, http://www.invitrogen.com/) using the Gateway protocol described by the manufacturer (Invitrogen). The hence obtained pENTR207::Δ*spAVR2*-Δ37 (N-terminal deletion-1), pENTR207::Δ*spAVR2*-Δ40, and pENTR207::Δ*spAVR2*-CTΔ11 (C-terminal deletion) plasmids were recombined into the binary vector CTAPi (Rohila et al., 2004) using the Gateway protocol (Invitrogen). The resulting plasmids, CTAPi::Δ*spAVR2*-Δ37, CTAPi::Δ*spAVR2*-Δ40, and CTAPi::Δ*spAVR2*-CTΔ11, were used for agroinfiltration as described below.

To generate the constructs used for yeast-two hybrid experiments, the *AVR2* ORF, lacking the sequence encoding the signal peptide, was amplified using primer FP1873 and FP1874. As template the *AVR2* gene in CTAPi was used (Houterman et al., 2009). The obtained product, carrying *Nco*I and *Eco*RI restriction sites, was cloned into the pAS2-1 and pACT-2 (Clontech) vectors digested with the same restriction enzymes.

For co-immunoprecipication experiments binary vectors containing Avr2 were created. *Xba*I and *Bam*HI restriction sites flanking the Δsp*AVR2* coding sequence were introduced by PCR with primers FP2525 and FP2274 using CTAPi:*:*Δsp*AVR2* as template (Houterman et al., 2009). The obtained product was sub-cloned into the vector SLDB3104 (Tameling et al., 2010) between the *Xba*I and *Bam*HI restriction sites to generate SLDB3104::Δsp*AVR2.* In the resulting plasmid Avr2 is fused to a C-terminal hemagglutinin (HA) and streptavidin-binding peptide (SBP) tag. All PCR primers were purchased from MWG (http://www.mwg-biotech.com), and sequences of all plasmids were confirmed by sequence analysis.

### Protein extraction and immunoblotting

Infiltrated *N. benthamiana* leaves were harvested and pooled 24 h after agroinfiltration, and snap-frozen in liquid nitrogen. After grinding the tissue with a mortar and pestle, it was allowed to thaw in 2 ml protein extraction buffer per gram of tissue [25 mm Tris pH 8, 1 mm EDTA, 150 mm NaCl, 5 mm DTT, 0.1% NP-40, 1× Roche complete protease inhibitor cocktail (http://www.roche.com) and 2% PVPP]. Extracts were centrifuged at 12 000 *g*, 4°C for 10 min, and the supernatant was passed over four layers of Miracloth (http://www.calbiochem.com/miracloth) to obtain a total protein lysate. 40 μL samples were mixed with Laemmli sample buffer, and equal amounts of total protein were run on 13% SDS–PAGE gels and blotted on PVDF membranes using semi-dry blotting. Skimmed milk powder (5%) was used as a blocking agent. A 1:3000 dilution of anti-GFP antibody (VXA6455, Invitrogen), or 1:8000 dilution of anti-tandem affinity purification (TAP) tag antibody (PAP, P1291, Sigma P1291) linked to horseradish peroxidase were used. The secondary antibody goat-anti-rabbit (P31430, Pierce) was used as a 1:5000 dilution. The luminescent signal was visualized by ECL using BioMax MR film (Kodak, http://www.kodak.com).

For mass spectrometry analysis, protein extracts were spun for 10 min at 12,000g, and 1 ml supernatant was added to 100 μl bed volume of Streptavidin Sepharose High Performance beads (GE Healthcare). Protein extracts were incubated in a rotator for 3 h at 4°C, and washed four times with immunoprecipitation buffer (25 mM Tris-HCl, pH 7.5, 1 mM EDTA, 150 mM NaCl, 10% glycerol, and 5 mM DTT, and 0.15% Nonidet P-40). Elution was performed twice with two bed volumes of washing buffer containing 4 mM D-biotin (Sigma-Aldrich). 400 μl eluted fractions were pooled and precipitated with trichloroacetic acid. Pellets were washed with 100% acetone at −20°C. 40 μl samples were mixed with Laemmli sample buffer for MS and loaded on 12% SDS-PAGE gels cased in Hoefer Might Small SE250 mini gel equipment (Amersham Biosciences, AB, Uppsala). After gel electrophoreses Coomassie PageBlue™ (Fermentas) staining was used to visualize the proteins.

### Mass spectrometry

The protein bands corresponding to the mass of the expected Avr2 monomer and dimer were sliced from the Coomassie stained gel. In-gel digestion was performed as described by Rep et al. (2002). The peptides obtained after the digestion were analyzed by MALDI-TOF/TOF MS as described by Krasikov et al. (2011). Acquired spectra were then searched with Mascot (Matrix Science, UK) against a *Fol* database. The *Fol* protein database used for the analysis was obtained from Fusarium Comparative Genome website (http://www.broadinstitute.org/annotation/genome/fusarium_group/MultiHome.html) and supplemented by adding the sequences of known Six proteins that are not annotated in the public database. To identify the plant proteins, all spectra were also searched against a custom Solanaceae EST database from plant-assembled transcripts (http://plantta.jcvi.org/).

### Yeast two-hybrid

The matchmaker GAL4 two-hybrid system and yeast strain PJ694a were used for analyzing protein interactions. Yeast transformation was performed using lithium-acetate and polyethylene glycol 3350 as described (Gietz and Woods, 2002). Eight colonies were picked and transferred from the MM-WL plates, lacking Trp and Leu, to a fresh MM-WL plate and incubated for 5 days at 30°C. Next, one colony per combination was re-suspended in 25 μl 0.9 % NaCl and 6 μl was spotted on MM-WL, MM-HWL, MM-AWL, and MM-HWL plates containing 3 mM 3-amino-1,2,4-triazole. After 5 days incubation at 30°C, the plates were checked for growth and photographed.

### Co-immunoprecipication

For Co-IP experiments, total proteins were extracted from *N. benthamiana* leaves, as described above, 36 h after infiltrating with *A. tumefaciens* GV3101 containing either SLD::Δsp*AVR2*-HASBP or pGWB451::Δsp*AVR2* or a mixture of both *A. tumefaciens* strains. Immunoprecipication was performed as described above. A portion of the supernatant was reserved as input sample. 20 μl immunoprecipicated samples and 40 μl input samples were resuspended in 1× SDS-PAGE loading buffer and loaded on 12% SDS-PAGE gels. Next, the gels were subjected to immunoblotting using anti-HA peroxidase at dilution ratio 1:3000 (clone 3F10; Roche), and anti-GFP at dilution ratio 1:3000 (Invitrogen, VXA6455).

### Confocal microscopy

Confocal microscopical analysis was performed with an LSM510 (Zeiss, Germany). Excitation of GFP was done at 488 nm with an Ar-ion laser and emission was captured with a 505–530 nm pass filter. Excitation of RFP occurred at 543 nm with a HeNe laser. The 590–620 nm filter captured emission. To monitor co-localization RFP was excited at 543 nm and GFP at 488 nm or YFP at 514 nm. GFP and YFP emission was captured with a 505–530 nm filter and RFP with a 565–615 nm filter. Images were scanned eight times.

### *Agrobacterium*-mediated transient transformation of *Nicotiana benthamiana*

*A. tumefaciens* strain GV3101(pMP90) (Koncz and Schell, 1986) was transformed with binary constructs as described previously (Takken et al., 2004). Agrobacterium-mediated transient transformation was performed according to methods described by Ma et al. (2012). Briefly, the agrobacteria were grown to an absorbance of 0.8 at 600 nm in LB-mannitol medium (10 g l^−1^ tryptone, 5 g l^−1^ yeast extract, 2.5 g l^−1^ NaCl, 10 g l^−1^ mannitol) supplemented with 20 μm acetosyringone and 10 mm MES pH 5.6. Cells were pelleted by centrifugation at 4000 g at 20°C for 20 min and then resuspended in infiltration medium (1× MS salts, 10 mm MES pH 5.6, 2% w/v sucrose, 200 μm acetosyringone). Infiltration was done in *N. benthamiana* leaves at an absorbance of 0.1 (for *I-2* constructs) or 0.5 (for *AVR2* constructs) of 4–5-weeks-old plants.

### Plasmolysis

For plasmolysis, a plasma membrane marker labeled with YFP (ZmHVR-YFP) (Ma et al., 2012) and Avr2-RFP were co-infiltated in *N. benthamiana* leaves. Two days after infiltration, small infiltrated leaf pieces were collected and treated with 800 mM mannitol for 30 min to induce plasmolysis. Subsequently, the pieces were mounted in 30% glycerol on a glass slide for microscopy.

## Results

### *AVR2* is predominantly expressed in xylem-colonizing fungal hyphae

**Figure 1:**
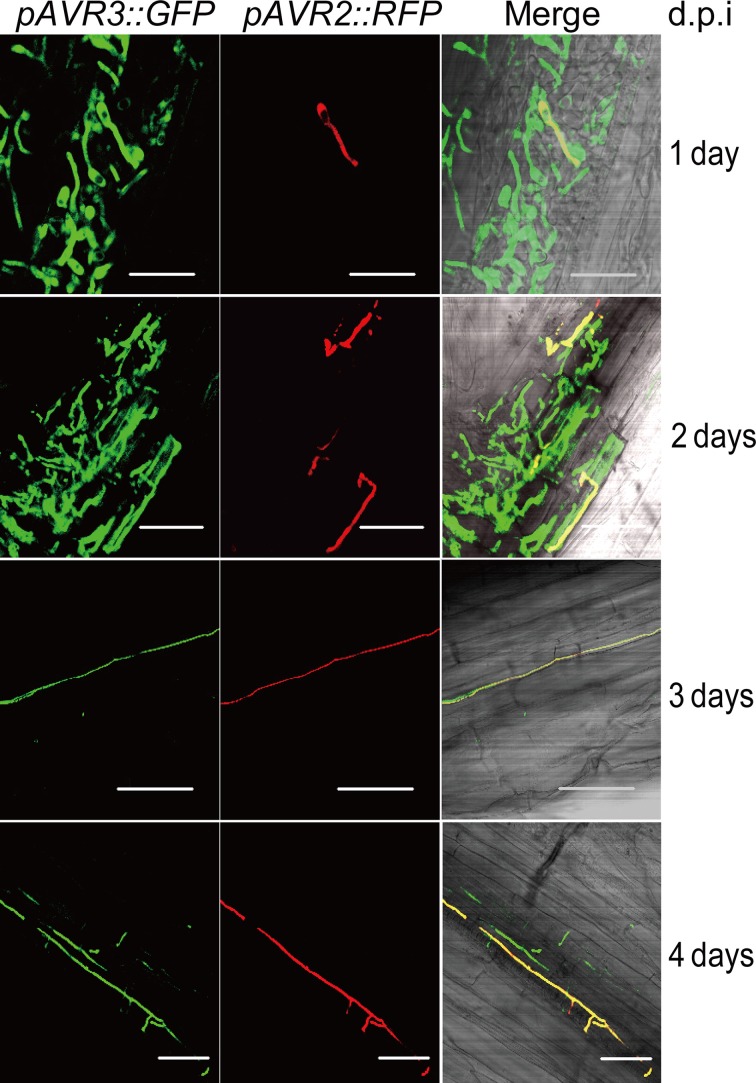
**Expression of p*AVR2:RFP* p*AVR3:GFP* in *Fol* colonizing tomato roots.** RFP and GFP fluorescence was visualized using confocal microscopy. Imagines are depicted as separate red and green channels and as a merged figure. Ten-days-old tomato seedlings were inoculated with a *Fol* spore suspension and roots were analyzed at different time points post inoculation. One day after inoculation germinating spores can be found on the surface of tomato roots. Two days after inoculation hyphae have penetrated the epidermis and start to grow between the cortical cells. Three days after inoculation hyphae are growing between cortical cells. After three days, hyphae grow inside the xylem vessels. White scale bars represent 25 μm.

To determine at which stage of infection *AVR2* is expressed, a *Fol* strain carrying an *AVR2*-promoter-reporter construct was created. mRFP was used as reporter and the p*AVR2:RFP* construct was transformed into a *Fol*-p*AVR3:GFP* strain. In this strain the coding sequence for Avr3 has been replaced by *GFP* encoding Green Fluorescent Protein (Van Der Does et al., 2008). The advantage of employing the *Fol*-p*AVR3:GFP* strain is that GFP can be used to monitor the growth of *Fol* in roots, as the *AVR3* gene is specifically expressed inside roots (Van Der Does et al., 2008). The p*AVR2*:*RFP* construct was designed to facilitate its integration into the *AVR2* locus by homologous recombination (see Materials and Methods) to ensure expression from the native locus, avoiding position effects. The double transformant was used to inoculate ten-days-old tomato seedlings grown hydroponically (Van Der Does et al., 2008). Expression of both reporter genes was studied in a time-course analysis by visualizing the RFP and GFP signals in inoculated roots using confocal microscopy. Figure 1 shows that, at one-day post inoculation, only few of the germinated spores penetrating the roots display RFP florescence (*AVR2* promoter activity), whereas a GFP signal (*AVR3* promoter activity) is present in many germinating spores penetrating the roots. This observation confirms the earlier finding that expression of *AVR3* is induced upon contact with tomato roots (Van Der Does et al., 2008), and shows that in the majority of hyphae that colonize the root cortex *AVR2* is not expressed. Two days after inoculation an RFP signal was still only detectable in a very limited number of hyphae or spores (Figure 1). At three days after inoculation red fluorescence was visible in some of the hyphae growing between the cortical cells (Figure 1), but the majority of the green fluorescent hyphae did not show red fluorescence. At stages later than three days after inoculation, RFP and GFP double fluorescent fungal hyphae were frequently found growing inside xylem vessels (Figure 1), demonstrating that *AVR2* is highly expressed at this stage of infection. However, still not all hyphae express both genes at this stage and frequently hyphae were observed that contained only GFP (or, sometimes, RFP). From these observations we conclude that upon contact with tomato roots and during early stages of infection, expression of *AVR3* precedes that of *AVR2.* From three days after inoculation and onward high expression of both *AVR3* and *AVR2* was found in hyphae growing in the xylem vessels of tomato roots.

### Avr2 localizes to the cytosol and nucleus of plant cells

**Figure 2:**
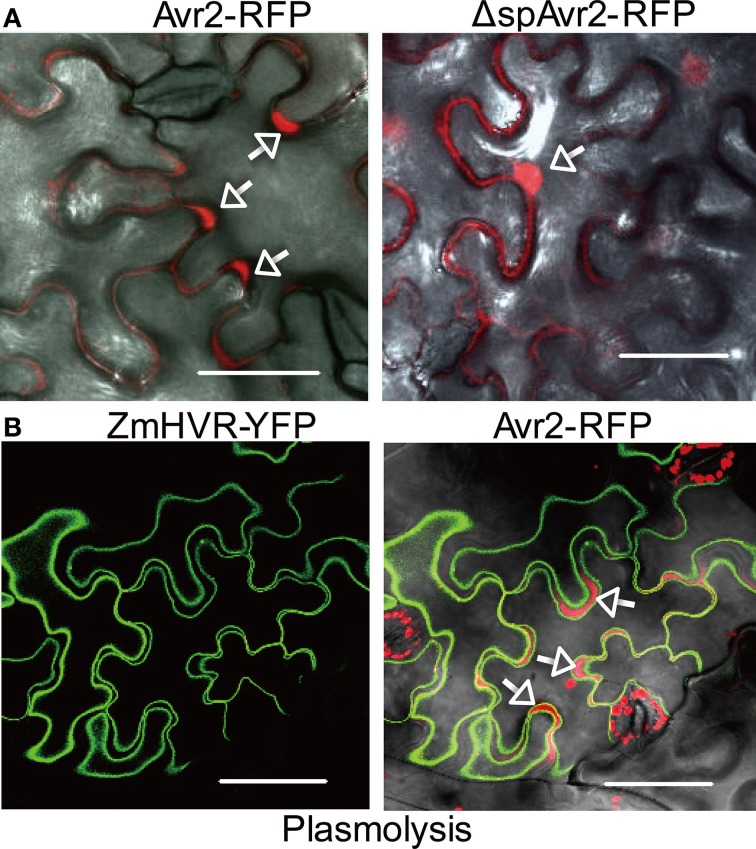
**Avr2 localizes in the cytosol and nucleus of *N. benthamiana* cells after agroinfiltration. (A)** Confocal images of mesophyll cells in *N. benthamiana* leaves 36 h after agroinfiltration with Avr2-RFP or ΔspAvr2-RFP lacking its signal peptide for secretion. White arrows indicate the apoplastic spaces. **(B)** Transient co-expression of Avr2-RFP and the plasma membrane marker ZmHVR-YFP in epidermal cells of *N. benthamiana* after plasmolysis. Avr2-RFP is clearly visible in the apoplastic spaces (arrows) that are enlarged due to plasmolysis. The white scale bars represent 25 μm.

To examine the localization of Avr2 in plant cells, *A. tumefaciens* harboring either full length *AVR2* C-terminally fused to *RFP* or *AVR2* lacking its signal peptide (Δsp*AVR2*) fused to RFP, was infiltrated in *N. benthamiana* leaves. The localization of Avr2-RFP and ΔspAvr2-RFP was then examined by confocal microscopy. The red fluorescence originating from the wild-type Avr2-RFP fusion was mainly found in the apoplastic space (Figure 2A, left panel, arrows). To confirm an apoplastic localization, and to exclude the possibility that Avr2 was tethered to the plasma membrane or cell wall, the *AVR2-RFP* construct was co-expressed with a plasma membrane marker (ZmHVR-YFP) and the plant cells were plasmolysed before microscopical analysis. As shown in Figure 2B, the yellow fluorescence from the ZmHVR-YFP protein is specifically localized at the plasma membrane flanking the diffuse red signal from Avr2-RFP, confirming the apoplastic localization of the latter (Figure 2B, right panel, arrows). The dispersed RFP signal suggests that Avr2 is secreted into the apoplast and diffuses between the plant cells. In contrast, the ΔspAvr2-RFP protein lacking its signal peptide localized inside the plant cells. Here the fusion protein was found in the cytosol and the nucleus (Figure 2A, right panel, arrow). The nuclear localization of Avr2 can clearly be seen by the exclusion of the fluorescent protein from the nucleolus—more easily visible using a GFP-tagged Avr2 protein.

### Nuclear localized Avr2 is required to trigger I-2-dependent cell death in *N. benthamiana*

**Figure 3:**
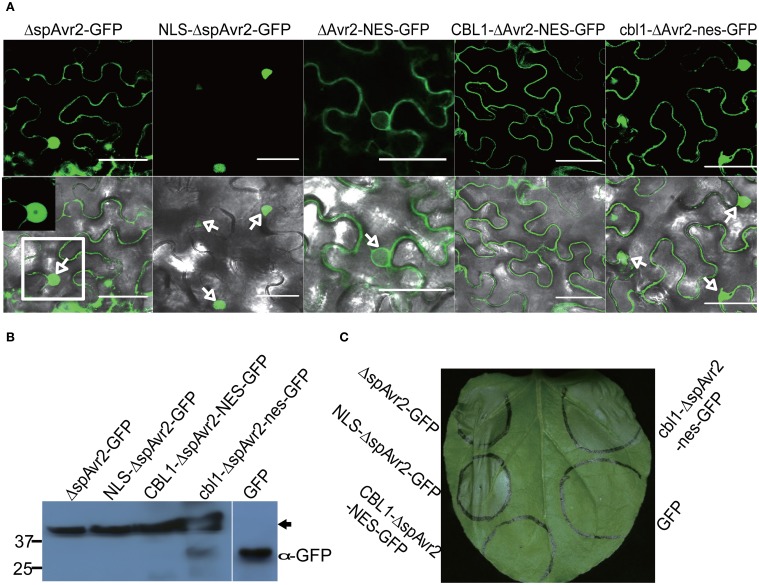
**Nuclear localization of Avr2 is required to trigger *I-2*-dependent cell death in *N. benthamiana* leaves. (A)** Confocal image of ΔspAvr2-GFP, NLS-ΔspAvr2-GFP, ΔspAvr2-NES-GFP, CBL1-ΔspAvr2-NES-GFP, and cbl1-ΔspAvr2-nes-GFP in *N. benthamiana* leaves 36 h after agroinfiltration. Arrows indicate the nucleus, top GFP channel, bottom GFP and bright field channels merged. **(B)** Immunoblot analysis of GFP-fusion proteins accumulating *in planta* 36 h after infiltration. Blots were probed with an α-GFP antibody. The GFP alone control was used to assess the specificity of the antibody. Sizes in kDa are indicated on the left and an arrow indicates the 43 kDa band of the full-length protein. **(C)** Nuclear localization is required for effector-triggered cell death. *N. benthamiana* leaves were co-infiltrated with *A. tumefaciens* carrying ΔspAvr2:GFP, NLS-ΔspAvr2:GFP, CBL1-ΔspAvr2-NES:GFP, cbl1-ΔspAvr2-nes:GFP, or GFP alone with a strain carrying an *I-2* containing vector. A representative picture was taken three days after infiltration. Cell death is visible by tissue collapse of the infiltrated region. The white scale bars represent 25 μm.

To determine at which subcellular localization Avr2 is recognized by I-2, Δ*spAVR2* was fused to either a nuclear import signal (NLS) at its N-terminus, or a NES at its C-terminus. A NLS targets the protein to the nucleus whereas the NES translocates Avr2 from the nucleus to the cytoplasm, reducing its nuclear concentration (Kalderon et al., 1984). To examine whether the localization signals are functional, the Δ*spAVR2* variants were C-terminally fused to *GFP* and expressed in *N. benthamiana* leaves using agroinfiltration. At 36 h after infiltration, green fluorescence was imaged using confocal microscopy. As observed before, wild-type Avr2, lacking its signal peptide (Δsp) and fused to GFP, localized in both cytosol and nucleus (Figure 3A, arrow). NLS tagged Avr2 (NLS-ΔspAvr2-GFP) was only detected in the nucleus and not in the cytoplasm (Figure 3A, arrows). In contrast, the NES tagged Avr2 (ΔspAvr2-NES-GFP) protein was found in both the cytoplasm and the nucleus albeit at a lower concentration as the ΔspAvr2-GFP control (Figure 3A). So, although the NES translocates Avr2 from the nucleus it does not exclude nuclear entry. To further reduce the amount of Avr2 in the nucleus the NES-containing Avr2 protein was fused to a CBL1 (Myristoylation signal) motif at its N-terminus to tether it to the plasma membrane, preventing nuclear entry. As a negative control an Avr2 fusion with both a mutated CBL1 (cbl1) and a mutated NES was made. Both constructs were expressed in *N. benthamiana* leaves using agroinfiltration and green fluorescence was imaged using confocal microscopy. The CBL1 and NES tagged Avr2 (CBL1-ΔspAvr2-NES-GFP) protein was found exclusively at the plasma membrane and not in the nucleus (Figure 3A). The Avr2 variant carrying the cbl1 and nes signals (cbl1-ΔspAvr2-nes-GFP) displayed a distribution similar as ΔspAvr2. Immunoblotting showed that all ΔspAvr2-GFP fusion proteins accumulate at similar levels (Figure 3B). The majority of the proteins are intact, as bands were found at the expected size of ~43 kDa. For the CBL1-Avr2-NES-GFP and cbl1-Avr2-nes-GFP extracts also some smaller bands were observed, which could be the consequence of limited proteolytic cleavage (Figure 3B).

We next utilized the above-described constructs to assess whether the enforced relocalization of Avr2 affected its ability to trigger I-2-mediated cell death. Thereto *I-2* and the *AVR2* constructs carrying the various translocation signals were co-expressed in *N. benthamiana* leaves using agroinfiltration. Figure 3C shows that at approximately 36 h after co-infiltration nuclear localized NLS-ΔspAvr2-GFP triggered an *I-2*-dependent cell death response equivalent to that of ΔspAvr2. In contrast, CBL1-Avr2-NES-GFP, which is retained in the plasma membrane, was unable to activate *I-2* and induce cell death. Avr2 fused to the mutated (inactive) CBL1 and NES motifs triggered an *I-2* specific cell death response similar to that of the ΔspAvr2 protein, showing that the mere extension of Avr2 with these sequences did not interfere with its cell death inducing activity (Figure 3C). Co-expression of *I-2* with the *NES*-tagged *Avr2* induced a cell death response comparable to that of co-expression with *cbl1-Avr2-nes-GFP* protein and wildtype Avr2 (data not shown) consistent with the similar subcellular distribution pattern of the latter proteins. In summary, these results indicate that a nuclear localization of Avr2 is required to trigger *I-2*-dependent cell death.

### The N-terminal region of Avr2 is dispensable to trigger *I-2*-mediated cell death

**Figure 4:**
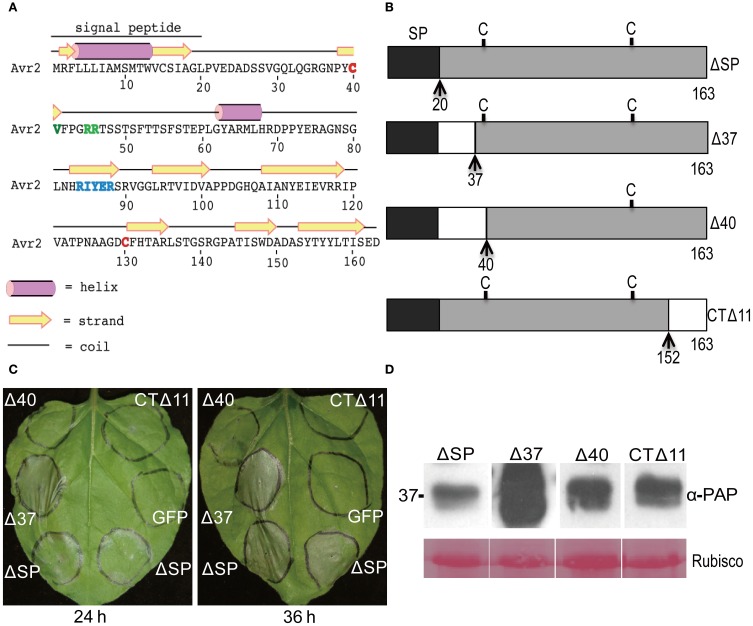
**A small N-terminal region of Avr2 is dispensable for *I-2*-dependent cell death. (A)** Secondary structure prediction of Avr2. The two cysteine residues are marked red. The three polymorphisms in *I-2*-breaking (*Fol* race 3) Avr2 variants are marked green. **(B)** Schematic diagram showing the Avr2 truncations. The signal peptide is shaded dark, C indicates a cysteine residue; N- and C-terminal truncation sites are indicated by dashed lines and arrows along with the corresponding amino acid number. **(C)** *N. benthamiana* leaves were co-infiltrated with *A. tumefaciens* cultures containing *AVR2* truncations and *I-2*. The left panel shows a leaf photographed 24 h after infiltration and the right panel a leaf photographed 36 h after infiltration. **(D)** Immunoblot of proteins extracted from agroinfiltrated *N. benthamiana* leaves expressing Avr2 truncations fused C-terminally to a TAP tag. Leaves were harvested 36 h after infiltration and analyzed by immunoblotting using an anti-PAP antibody recognizing the TAP tag (α-PAP). Ponceau S staining of Rubisco (lower panel) is shown as a measure of the amount of protein loaded in each lane. Sizes in kDa are indicated on the left.

To define the minimal region of Avr2 required to trigger *I-2*-dependent cell death, two N-terminally truncated and one C-terminally truncated Avr2 protein was generated. To assist demarcation of the Avr2 truncations, PSIPRED (Buchan et al., 2010) was used to predict the secondary structure of the Avr2 protein (Figure 4A). Based on this prediction, the following variants were constructed: Avr2Δ37, in which the first predicted random coil downstream of the signal peptide was deleted; Avr2Δ40, a slightly extended deletion that includes the first cysteine; and Avr2CTΔ11, which lacks the last predicted β-strand at its C-terminus (Figure 4B). Both wild type and the three variants were equipped with a C-terminal TAP-tag.

*A. tumefaciens* strains containing plasmids encoding these truncated *AVR2-TAP* constructs were co-infiltrated with an *A. tumefaciens* strain harboring *I-2* into *N. benthamiana* leaves. Expression of Δ*spAVR2-TAP* together with *I-2* served as a positive control, and *I-2* together with an *GFP* containing vector as a negative control. Compared to ΔspAvr2, Avr2Δ37 induced a much faster and stronger cell death response (Figure 4C). I-2-dependent cell death triggered by Avr2Δ37 was observed as early as 20–24 h after infiltration, whereas cell death induced by Avr2 did not appear until 10–12 h later. Expression of the two other truncated variants, Avr2Δ40 and Avr2CTΔ11, did not induce *I-2*-dependent cell death (Figure 4C). Immunoblotting analysis demonstrated that all truncated proteins accumulated at similar levels, except the Avr2Δ37 truncation that accumulated in much higher amounts (Figure 4D). Hence, the inability of Avr2Δ40 and CTΔ11 to trigger *I-2*-mediated cell death is not due to a lack of protein accumulation. The high accumulation of Avr2Δ37 might be correlated with its ability to trigger a faster and stronger cell death response. Based on these observations, we concluded that Avr2 can be functionally divided into two parts, a small N-terminal part that is not required for I-2-mediated cell death and a large C-terminal region that includes the two cysteines and is indispensable for this activity.

### Avr2 homodimerizes *in vivo*

**Figure 5:**
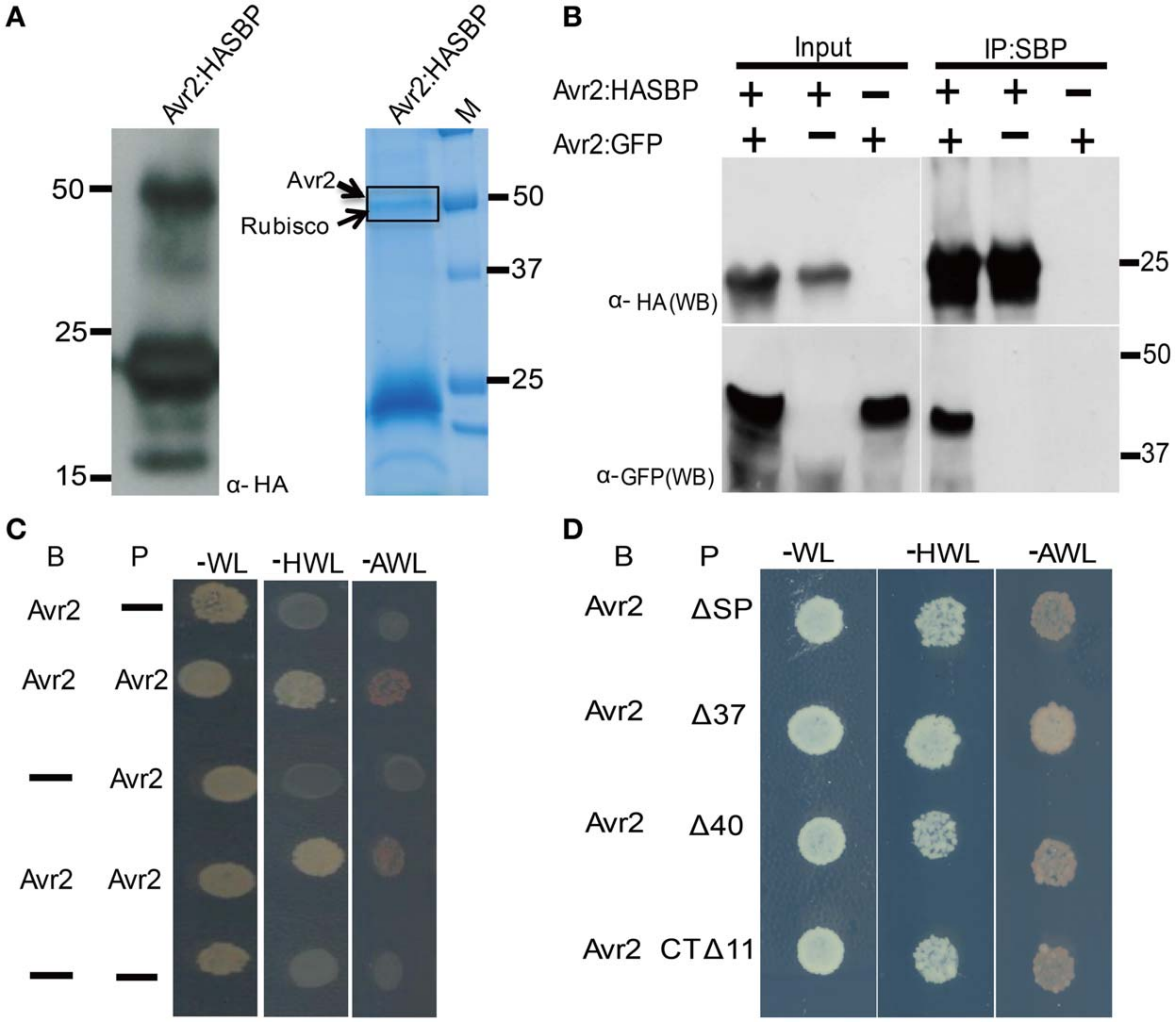
**Avr2 forms homodimers *in planta* and in yeast. (A)** Immunoblot probed with anti-HA showing that Avr2-HA-SBP is detected at the expected apparent molecular weight of ±25 kDa as well as in a ±50 kDa band. Avr2-HA-SBP was expressed in *Agrobacterium*-infiltrated *N. benthamiana* leaves and subsequently affinity-purified using the SBP tag. The purified protein was size separated using SDS-PAGE and the gel was stained with Colloidal Coomassie. The dashed rectangle indicates the section used for mass spectrometric analysis and the identified proteins are indicated on the left. Positions and sizes of the molecular weight marker are shown. **(B)** Immunoprecipitation of Avr2 from total plant protein extracts. Proteins extracted from *N. benthamiana* leaves expressing pairwise combinations of Avr2 C-terminally tagged with either HASBP or GFP. The fusion proteins were immunoprecipitated using streptavidin beads. Total extracted proteins (input) and immunoprecipitated proteins (IPs) were analyzed by immunoblotting by probing with either anti-HA (α-HA; upper) or anti-GFP (α-GFP; lower). Positions and sizes of protein mass makers are shown. **(C)** Growth of yeast strain pJ694a transformed with prey (P) constructs containing *AVR2* or empty vector (−) and bait (B) constructs containing *AVR2* or empty vector (−). All transformed yeasts could grow on minimal media lacking tryptophan and leucine (−WL) due to presence of the bait and prey plasmids. Only yeast containing both Avr2 as prey and bait was able to grow on selection plates lacking histidine, tryptophan and leucine (−HWL), and the more stringent selection medium lacking alanine, tryptophan and leucine (−AWL). Neither empty bait nor prey or Avr2 alone in combination with an empty vector could grow on the selection plates. **(D)** All Avr2 truncations interacted with wild-type Avr2 in yeast.

Immunobloting of agroinfiltrated *N. benthamiana* leaves expressing a C-terminally human influenza HA and streptavidin-binding peptide (SBP) double-tagged Avr2 fusion protein (Avr2-HASBP), frequently revealed an additional ~50 kDa band, i.e., twice the molecular mass of the Avr2-HASBP protein (Figure 5A). Since this larger product cross-reacted with the HA antibody it likely contains the Avr2-HASBP protein incorporated in a larger complex. To identify the constituents of this complex mass spectrometric analysis was performed. Avr2-HASBP containing complexes were affinity purified using SBP beads from a protein extract isolated from agroinfiltrated *N. benthamiana* leaves transiently expressing *AVR2-HASBP.* Next, the purified Avr2 protein complexes were size-separated on SDS-PAGE and the region corresponding to the ~50 kDa product was cut out from the gel (Figure 5A). The proteins in the slice were in-gel digested and subjected to mass spectrometric analysis. Four peptides matching to Avr2 and ten peptides corresponding to Rubisco were identified in the peptide list (data not shown). Since the latter is a likely contaminant, the absence of other proteins raised the possibility that Avr2-HASBP might homodimerize, giving rise to the 50 kDa product.

To confirm the ability of Avr2 to physically self-interact *in planta*, co-immunoprecipitation experiments were performed. Two different *AVR2* constructs were used that each carry a different epitope tag: HASBP or GFP. Following co-agroinfiltration of these constructs into *N. benthamiana* leaves, the Avr2-HASBP protein was pulled down using SBP affinity beads and co-purification of the other was assessed using its GFP tag. Figure 5B shows that GFP-tagged Avr2 co-precipitates with HASBP-tagged Avr2 when both genes were co-expressed in *N. benthamiana* leaves, demonstrating that Avr2 has the ability to multimerize. To further test this, a yeast-two hybrid experiment was conducted using Avr2 both as bait and prey. As shown in Figure 5C, Avr2 interacts with itself, as yeast transformed both with bait and prey plasmids harboring Avr2 grew on the selective-HWL and also on the more stringent-AWL medium. Yeast co-transformed with Avr2 and empty bait or prey plasmid was unable to grow on the selection plates (Figure 5C). Together, these data strongly suggest that Avr2 can form dimers *in planta* and in yeast.

To determine whether dimerization is correlated with the ability of Avr2 to activate I-2, the dimerization capacity of the three truncated Avr2 variants (Figure 4) was examined using yeast two-hybrid assays. As shown in Figure 5D all constructs supported growth on selective-HWL and -AWL medium, suggesting that the central region of Avr2 of 104 amino acids (aa 40-144) is sufficient for dimerization. Since Avr2Δ40 and Avr2CTΔ11 are not capable of activating I-2 it can be concluded that dimerization of the central region alone is not sufficient to induce *I-2*-mediated cell death.

## Discussion

Expression of *AVR2* was rarely observed during early stages of infection when the fungus colonizes the epidermis and invades the roots to grow between the cortical cells (Figure 1). However, during later stages of infection when the fungus colonizes the xylem vessels *AVR2* expression could readily be detected (Figure 1). This expression pattern differs from that of *AVR3* (*SIX1*), which was found to be expressed early upon infection and green fluorescence can be visualized already one-day post inoculation. The *AVR3* gene continues to be expressed during later stages of infection [(Van Der Does et al., 2008) and Figure 1]. Notably, at these later stages many fungal hyphae could be observed that express both genes, but also hyphae were found that express only one of the two genes. The latter is surprising since previous studies showed that expression of most *Six* genes, including *AVR3* (*SIX1*), and *AVR2* (*SIX3*), depends on the presence of the same transcription factor, Sge1 (Six Genes Expression 1) (Michielse et al., 2009). The dissimilar expression of *AVR2* with *AVR3* indicates that expression of these genes is not solely regulated by Sge1, but is likely also controlled by other factors. It will be interesting to analyse the expression profile of other effector genes during infection and to compare these to *AVR3* and *AVR2* to identify whether they are controlled in similar fashion. The relatively late expression of *AVR2* suggests that *I-2*-mediated resistance occurs relative late in infection, e.g., when the fungus colonizes the xylem vessels. *I-2*-mediated resistance acting in the xylem tissues is in agreement with (1) the expression of *I-2* in the vasculature and its lack of expression in cortical root cells (Mes et al., 2000), (2) the presence of the Avr2 protein in the xylem sap of tomato (Houterman et al., 2009), and (3) the observation that in an *I-2* plant *Fol* is able to colonize the cortical root cells and to reach the xylem vessels which it can colonize to an extent (Rep, pers. communication).

Deletion of the N-terminal 17 amino acids (Δ37) of Avr2 did not impair its ability to trigger *I-2*-dependent cell death. Actually, upon agrotransformation the truncated protein induced a faster and stronger cell death response than full length Avr2 protein, which correlated with an increased accumulation of the truncated protein (Figures 4C and D). The mechanism underlying the higher accumulation of this truncated protein is unknown, but the truncated form resembles the shorter forms found in the xylem sap. On 2D protein gels of xylem sap from infected tomato plants Avr2 localizes in at least three spots ranging in size from 11 to 14 kDa. Mass spectrometric analysis of these spots revealed that the smallest form of Avr2 in xylem sap has a N-terminal deletion similar to that of Δ37 (Houterman et al., 2007). The removal of these 17 aa might be due to N-terminal processing by plant proteases in xylem sap. An extended deletion removing 20 amino acids at the N-terminus encompassing the first cysteine after the signal peptide (Δ40) completely abolished the ability of Avr2 to trigger *I-2*-dependent cell death (Figure 4C). Since there are two cysteine residues present in Avr2 (Figure 4A), it is possible that a disulfide bond is formed in the mature Avr2 protein. Deletion of the cysteine would disrupt this bond, potentially affecting protein structure. However, the protein apparently retains at least part of its fold, as the mutant is still able to interact with wild-type Avr2 in yeast. Alternatively, the cysteine at position 40 might be part of a motif that is required for I-2-mediated recognition. Support for this hypothesis is the observation that Avr2 variants from race 3 strains of *Fol* that overcome I-2-mediated resistance carry a mutation in one of three nearby residues; valine 41, arginine 45, or arginine 46 (Houterman et al., 2009). Possibly, these residues together form an epitope that is recognized by I-2. A C-terminal deletion also abolished *I-2*-mediated recognition, but retained the proteins' ability to interact with wild-type Avr2 in yeast (Figure 5D). Together, these data show that dimerization alone is insufficient to activate I-2, and that also the C-terminus contains sequences required for I-2-mediated recognition (Figure 5D). The central part that can dimerize contains the “RIYER” sequence motif that was identified by Kale and co-workers as an “RXLR-like” motif that could be involved in entry of this effector in plant cells (Kale et al., 2010). A truncated Avr2 protein, consisting of the N-terminal half of the protein containing this domain, was taken up by soybean root cells whereas various RIYER mutants were not (Kale et al., 2010). In many oomycete effectors mutations in the conserved RxLR motif abolish their uptake by host cells (Grouffaud et al., 2008). One proposed function for the RxLR motif is binding to phosphatidylinositol-3-phosphate (PI3P) present on the outer surface of the plant plasma membrane, enabling vesicle-mediated endocytosis (Kale et al., 2010). An alternative function for the RXLR motif of an oomycete effector was recently proposed for AVR3a from *Phythophthora infestans* in which this region is required for homodimerization (Boutemy et al., 2011; Wawra et al., 2012). Whether the RIYER motif in Avr2 is also required for dimerization awaits elucidation of its 3D protein structure. Solving the structure will not only aid identification of surface localized residues involved in homodimerization, but could also reveal residues that mediate interaction with plant proteins.

Using a heterologous expression system, we demonstrated that nuclear localization of Avr2 is required to trigger an *I-2*-dependent cell death response (Figure 3). Unfortunately, the subcellular localization of I-2 is unknown and its determination is hampered by the lack of sensitive antibodies and the loss of function of tagged I-2 variants (Tameling et al., 2002). A truncated I-2 protein, lacking its LRR domain, localizes in both the nucleus and the cytosol when expressed via agroinfiltration in *N. benthamiana* (manuscript in preparation). In addition, a potential NLS (RKHK) has been predicted in the CC domain of I-2 (Simons et al., 1998). These observations imply that I-2 could also be localized in the nucleus.

Proximity of an NLR to its recognized effector(s) is a likely prerequisite for its activation. Recent examples are Arabidopsis RPM1 that initiates signaling at the plasma membrane where its effectors AvrRpm1 and AvrB reside (Gao et al., 2011). Likewise, the potato R3a protein is activated only when it colocalizes with its cognate *Phytophtora infestans* effector Avr3a^KI^ at endosomal compartments (Engelhardt et al., 2012). Finally, the tobacco Rx protein needs to co-localize with the viral effector in the cytoplasm to become activated (Slootweg et al., 2010). The different sites for NLR activation could reflect the surveillance of diverse effector activities, which would imply a nuclear function for Avr2. Whether I-2 resistance signaling also requires its nuclear location remains to be investigated. A growing body of evidence suggest that nuclear or a nucleocytoplasmic localization for at least some R proteins, such as tobacco N, potato Rx, and barley Mla10 is essential for proper immune signaling (Burch-Smith et al., 2007; Slootweg et al., 2010; Tameling et al., 2010; Bai et al., 2012; Heidrich et al., 2012) [reviewed by Deslandes and Rivas (2011), Rivas (2012)]. Interestingly, the *P. syringae* effector AvrRps4 was found to trigger compartment-specific immune responses in which nuclear localized AvrRps4 triggers RPS4-dependent resistance and cytoplasmic AvrRps4 induces cell death, implying that cell death and resistance signaling are independent processes (Heidrich et al., 2011). The barley resistance protein MLA10 also displays compartment-specific immunity; the nuclear pool being involved in resistance and the cytoplasmic pool in triggering cell death (Bai et al., 2012). It will be interesting to determine whether Avr2-mediated cell death and resistance also require different locations for I-2 or whether both immune responses originate from the plant nucleus.

### Conflict of interest statement

The authors declare that the research was conducted in the absence of any commercial or financial relationships that could be construed as a potential conflict of interest.

## Appendix

**Table A1:** **Primers used in this study**.

**Number**	**Sequences**
FP2708	GCGGTACCACTAGTTTCTGTGGCAGTTCCCCT
FP2663	CCCGGTACCCAGTCCCCACACAGTATTCTTTC
FP1074	CCAGCCAGAAGGCCAGTTT
FP2706	CCACTAGTATGGCCTCCTCCGAGGAC
FP2707	CGACTAGTAGATCTTTAGGCGCCGGTGGAGTGGCGG
FP3478	CAAAGGCAGCAAAAGAATTTCCATATTGCGTGTTTCCCGGCCGCCGCACGT
FP3482	AGAGGAGGAAGTTGAAGATCCTCTGAGATAGTAAGATAGTAGGTATAACT
FP3481	GCGTCTAGAATGGCCAGCTTCCACTCAAAGGCAGCAAAAGAATTTCCATA
FP3485	GCGTCTAGAAAGAGTGGCTCTTTCAGCAGGAGGGGCTTGAAGATCCTCTG
FP3480	CAAAGGCAGCAAAAGAATTTCCATATTGCGTGTTTCCCGGCCGCCGCACGT
FP3484	GCAGGAGGGGCTTGAAGATCCTCTGAGATAGTAAGATAGTAGGTATAACT
FP2959	CCTCTAGATTGCTCACCTAAGAAGAGAAAGGTTGGAGGAC
FP2222	CGCGCGAGCTCTTATTTGTATAGTTCATCCA
FP3479	GCGTCTAGAATGGGCTGCTTCCACTCAAAGCAGCAAAAGAATTT
FP3483	GCGTCTAGAAAGAGTAAGTCTTTCAAGAGGAGGAAGTTGAAGATC
FP1749	AAAAAGCAGGCTGGATGCCTGTGGAAGATGCCGATTCATC
FP1751	AGAAAGCTGGGTATCCATCCTCTGAGATAGTAAGATAG
FP2684	AAAAAGCAGGCTCTATGCCATATTGCGTGTTTCCCGGCCG
FP2699	AAAAAGCAGGCTCTATGGTGTTTCCCGGCCGCCGCACG
FP2685	AGAAAGCTGGGTAAGCGTCGGCATCCCAACTGATTGTG
FP872	GGGGACAAGTTTGTACAAAAAAGCAGGCT
FP873	GGGGACCACTTTGTACAAGAAAGCTGGGT
FP1873	AAACCATGGAAGATGCCGATTCATC
FP1874	AAAGAATTCAATCCTCTGAGATAGTAAG
FP2525	CGCTCTAGAATGCCTGTGGAAGATGCCGAT
FP2274	GCGGGATCCTCCATCCTCTGAGATAGTAAG
